# From model to man: Understanding Tregs' dual role in MASLD

**DOI:** 10.1016/j.jhepr.2025.101619

**Published:** 2025-10-09

**Authors:** Janine Dywicki, Laura Elisa Buitrago-Molina, Anna K. Baumann, Ana C. Davalos-Misslitz, Celina M. Hendriks, Katharina L. Hupa-Breier, Maren Lieber, Jerome Schlue, Matthias Blüher, Heike Bantel, Christine S. Falk, Christian Koenecke, Freya Wellhöner, Benjamin Heidrich, Michael P. Manns, Fatih Noyan, Heiner Wedemeyer, Richard Taubert, Elmar Jaeckel, Matthias Hardtke-Wolenski

**Affiliations:** 1 Dept. of Gastroenterology, Hepatology, Infectious Disease and Endocrinology, Hannover Medical School, Germany; 2 Inst. of Medical Microbiology, University Hospital Essen, University Duisburg-Essen, Essen, Germany; 3 Inst. of Pathology, Hannover Medical School, Germany; 4 Dept. of Endocrinology and Nephrology, University of Leipzig, Leipzig, Germany; 5 Integrated Research and Treatment Center Adiposity Diseases, University of Leipzig, Leipzig, Germany; 6 Inst. of Transplant Immunology, IFB-Tx, Hannover Medical School, Germany; 7 Inst. of Immunology, Hannover Medical School, Germany; 8 Dept. of Hematology, Oncology, Hemostaseology, and Stem Cell Transplantation, Hannover Medical School, Germany; 9 Dept. of Liver Transplantation, Multi Organ Transplant Program, University Health Network, Toronto, University of Toronto, Canada

**Keywords:** Adaptive immunity, Clonal T cell expansion, Foxp3, Immunometabolism, Kupffer cells, MASH, Monocyte-derived macrophages, Regulatory T cells, T-cell receptor repertoire

## Abstract

**Background & Aims:**

Metabolic Dysfunction-associated Steatotic Liver Disease (MASLD) affects around 30% of the world's population and is often associated with metabolic conditions such as obesity, diabetes and hypertension. Approximately 10-20% of metabolic dysfunction-associated steatotic liver disease cases progress to metabolic dysfunction-associated steatohepatitis (MASH), which significantly increases the risk of liver cancer. While intrahepatic immune responses involving CD4+ and CD8+ T cells are potential therapeutic targets, their role in the pathogenesis of MASH is not fully understood. Regulatory T cells (Tregs), whose involvement has been controversial, require further investigation.

**Methods:**

In this study, we investigated the impact of adaptive immunity on MASH using a high-fat/high-carbohydrate diet (HF-HCD) model in wild-type and *Rag2*-deficient C57BL/6 mice, supplemented with human liver samples.

**Results:**

Our results showed that HF-HCD induced glucose intolerance and MASH, independent of adaptive immunity. Surprisingly, HF-HCD increased intrahepatic Treg numbers and the Treg/Teff ratio but did not alleviate the disease; instead, this increase correlated with greater disease severity. With progressing metabolic inflammation, an increased proportion of Tregs also expressed IL-17, which correlated with more severe liver pathology.

**Conclusions:**

The observed expansion of Tregs and the resulting increase in the Treg/Teff ratio did not protect against MASH but correlated with more severe disease in both mice and humans, consistent with a pro-inflammatory shift towards IL-17-producing (T_H_17-skewed) cells. These findings highlight the complex role of adaptive immunity in MASH progression and provide potential targets for future immunomodulatory therapies.

**Impact and implications:**

Diet-induced steatohepatitis can develop without adaptive immunity, yet IL-17A-expressing Foxp3^+^ T regulatory cells (Tregs) and CD8^+^ T cells markedly amplify hepatocellular injury and fibrosis. By separating initiating (macrophage-driven) from amplifying (adaptive) immune signals, our study refines current pathogenic models and provides hepatologists and immunologists with stage-specific therapeutic targets. For clinicians and trial designers, the data indicate that stabilizing Tregs or selectively dampening pro-inflammatory T-cell subsets could complement metabolic interventions in metabolic dysfunction-associated steatotic liver disease/steatohepatitis, whereas indiscriminate Treg expansion might be counter-productive. Because the conclusions are drawn from a murine model, translation to humans will require validation in patient-derived tissues and non-invasive immune biomarkers.

## Highlights

• Tregs accumulate in HF-HCD–induced murine MASH, and their numbers correlate with NAS severity.
• A subset of these intrahepatic Tregs acquires IL-17 expression, which increases with disease progression in mice.
• Human MASH biopsies likewise display increased lobular Treg density that correlates with NAS.
• Adaptive immunity amplifies, but is not required to initiate, MASH; *Rag2*^-/-^ mice develop milder inflammation.
• The HF-HCD model mirrors key immune features of early human MASH, highlighting Tregs as potential therapeutic modulators.

## Graphical abstract

**Figure:**
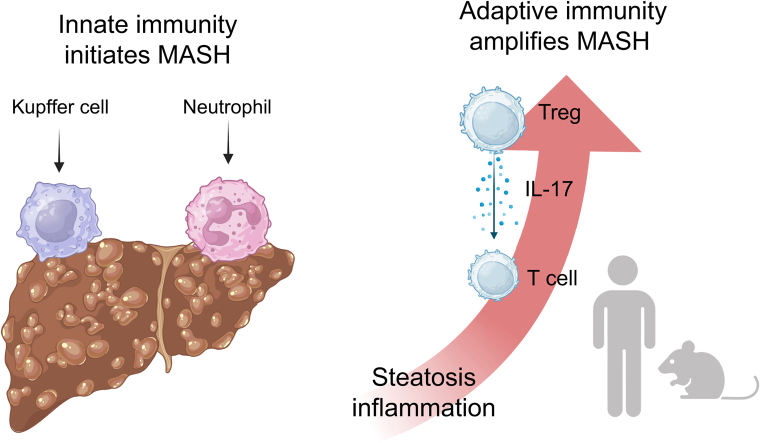

## Introduction

Metabolic dysfunction-associated steatotic liver disease (MASLD) affects approximately 30% of the global population.^1^ MASLD is associated with common metabolic comorbidities, such as obesity, insulin resistance, type 2 diabetes mellitus, hyperlipidemia, hypertension, and metabolic syndrome and 10-20% of patients with MASLD develop metabolic dysfunction-associated steatohepatitis (MASH) that can progress to hepatocellular carcinoma (HCC).^1^^,^^2^ Therefore, although most MASLD patients die from cardiovascular events rather than liver-related causes, MASLD is a potentially lethal disease.^1^^,^^2^ MASH is the second most common cause of liver transplantation worldwide and is expected to become the leading indication for liver transplantation in North America.^3^

Currently, there is no established therapy for MASH, beyond lifestyle changes and weight loss. Because MASH is caused by a chronic state of inflammation, modulation of intrahepatic immune responses may be a future therapeutic option. The lobular inflammatory infiltrates in MASLD/MASH are mixed and consist of neutrophils, macrophages, and lymphocytes. While the role of innate immunity, particularly pro-inflammatory macrophages, has been extensively demonstrated in mice and humans,^4^^,^^5^ increasing evidence points to the role of adaptive immunity in the pathophysiology of MASH.[6], [7], [8] CD4^+^ T cells likely play an important role in the development and progression of MASH, as increased numbers of intrahepatic T_H_17 cells have been reported in mice and patients with MASH.^6^^,^[8], [9], [10], [11] Recent evidence also suggests a role for CD8^+^ T cells and NKT cells in the pathogenesis of MASH, as MASH development was completely prevented in C57BL/6 *Rag1* and *ß2m* knockout in mice.^12^ Interestingly, CD8^+^ T-cell depletion led to the reversal of liver damage, while depletion of CD4^+^ T cells had no effect on MASH but increased the occurrence of MASH-induced HCC.^11^^,^^12^ Therefore, modulation of the adaptive immune response may be a double-edged sword, and further studies are necessary to clarify the role of different T-cell subsets in the pathogenesis and progression of MASH. In particular, the role of regulatory T cells (Tregs) remains to be elucidated, as both an increase and decrease in the number of these cells have been observed in diseased liver.[11], [12], [13]

In the present study, we investigated the role of adaptive immunity in a disease-relevant model of high-fat/high-carbohydrate diet (HF-HCD) feeding in wild-type and *Rag2*^*-/-*^ C57BL/6 mice. We found that the induction of glucose intolerance and MASH by HF-HCD was independent of adaptive immunity. Furthermore, diet-induced gut dysbiosis influenced the development of MASH more than adaptive immunity. However, HF-HCD induced the accumulation of Tregs in inflamed livers and increased the intrahepatic Treg/T effector cell (Teff) ratio. Unexpectedly, this increase did not correlate with disease improvement but with disease severity in mice and humans, demonstrating that adaptive immunity plays an important role in disease progression.

## Material and methods

### Mice

Animal care and experiments were performed in accordance with institutional and national guidelines. All animal experiments were performed according to the protocols approved by the Animal Welfare Commission of the Hannover Medical School and the Local Ethics Animal Review Board (Lower Saxony State Office for Consumer Protection and Food Safety, Oldenburg, Germany). Animals were purchased and maintained under specific pathogen-free conditions at the central animal facility of the Hannover Medical School. C57BL/6 *Rag2* ^*−/−*^ mice were bred in house. Six-to 8-week-old mice were fed a normal chow diet (NCD) from ssniff (Altromin standard diet # 1324 TPF, Altromin) or a HF-HCD (ssniff EF R/M D12330 mod. ∗/Surwit + 1% cholesterol, ssniff) with 45 g/L 55% fructose/45% sucrose (Sigma) in drinking water for 16 weeks. All *Rag2*^-/-^ and wild-type mice were inspected daily; none showed ruffled fur, weight loss >15%, splenomegaly or other signs of infection throughout the study.

For the intraperitoneal glucose tolerance test, 16-hour-fasted mice received 1 mg of glucose per gram of body weight. Blood glucose levels were measured in blood collected from the mouse tail vein using OneTouchUltra® strips (LifeScan).

### Patients

We retrospectively analyzed adult patients with biopsy-proven MASLD at Hannover Medical School. The patient data are summarized in Table S2. Patients were randomly selected according to the NAFLD activity score (NAS) to facilitate the analysis of the entire spectrum of histological disease severity. According to the current AASLD guidelines, the diagnosis of MASH requires the presence of both lobular inflammation and hepatocellular ballooning in addition to steatosis, whereas MASLD without MASH is defined as steatosis that does not fulfil these criteria.^14^

This study was approved by the local research Ethics Committee of the Hannover Medical School and University of Leipzig, and written informed consent was obtained from each patient.

### Immunohistological methods

Intrahepatic immunophenotyping of human liver biopsies was performed as previously described.^15^ We determined lobular and portal T-cell infiltration by encircling the liver lobules and portal tracts along the limiting plate and excluding the lumen of the veins, arteries, and bile ducts. We histologically analyzed intrahepatic infiltration of CD4^+^CD8^-^ (CD4^+^), CD8^+^CD4^-^ (CD8^+^), CD4^+^CD8^-^FOXP3^+^ (Tregs), and CD8^+^CD4^-^FOXP3^+^ (CD8^+^FOXP3^+^) cells. A second blinded observer assessed the FOXP3 expression. Treg detection via immunofluorescence of human FFPE tissue was recently validated using flow cytometry and epigenetic analysis.^15^ See supplementary CTAT table for full reagent and resource details.

### Serum analysis

Aspartate and alanine aminotransferase levels were determined by photometric enzymatic activity assays using an Olympus AU400 Chemistry Analyzer with serum, as previously described.^16^

Proteins in mouse sera were measured using the Olink® MOUSE EXPLORATORY panel∗ (Olink Proteomics AB, Uppsala, Sweden), according to the manufacturer's instructions, as previously described.^17^ The Proximity Extension Assay technology used for the Olink protocol has been well described and enables the simultaneous analysis of 92 analytes (further details can be found in the supplementary materials and methods).

### Histological assessment of liver biopsies

Murine liver tissue was fixed in formalin and embedded in paraffin. FFPE sections (5 μm) were prepared for H&E, Oil red O (triglyceride), and Sirius Red (collagen) staining. After staining, murine and human liver sections were examined in a blinded manner by a pathologist using the approved modified NAS system for nonalcoholic steatohepatitis and liver fibrosis.^14^^,^^18^ Mice were classified as having MASLD, borderline MASH, or MASH according to the NAS.

### Flow-cytometric definition of innate hepatic immune subsets

Single-cell suspensions of mouse livers were prepared by collagenase IV/DNase digestion, filtration (70 μm), differential centrifugation to remove parenchyma, erythrocyte lysis, and Fc-block as described before.^13^^,^^19^ Live CD45^+^ singlets were gated hierarchically (Table 1).

**Table 1:** Gating strategy.

#	Population	Phenotypic gate	Notes
1	Kupffer cells (KC)^20^	F4/80^high^ CD11b^neg/low^	Resident liver macrophages, Fig. 3
2	Monocyte-derived macrophages (MoMF)^20^	F4/80^high^ CD11b^high^	Subdivided by Ly6C into Ly6Chigh (M1-like) and Ly6Clow (M2-like), Fig. 3
3	Neutrophils	CD11b^+^ Ly6G^+^ Ly6C^low^	Fig. 3
4	Plasmacytoid dendritic cells (pDC)	CD11c^+^ CD11b^-^ Ly6C^+^	Fig. S1
5	Conventional DC type 1 (cDC1)	CD11c^+^ CD11b^-^ Ly6C^−^	Fig. S1
6	Conventional DC type 2 (cDC2)	CD11c^+^ CD11b^+^ Ly6C^−^	Fig. S1
7	Inflammatory monocytes	CD11c^+^ CD11b^+^ Ly6C^high^	Fig. S1
8	NK1.1+ innate lymphocytes^4^^,^^21^	NK1.1^+^ CD3^-^	Separated into iNK (CD11b^low^), mNK (CD11b^high^) and ILC1 (CD11b^-^), Fig 3

### Real-time PCR

Nucleic acid isolation, cDNA synthesis, pre-amplification, quantitative reverse-transcription PCR from mouse tissue, and bioinformatics analyses were performed as previously described.^17^ In brief, total RNA was isolated from frozen mouse liver samples, quantified using a NanoDrop 1000 spectrophotometer, and reverse-transcribed into cDNA. Pre-amplification of cDNA and quantitative reverse-transcription PCR were performed in 48.48 Dynamic Array IFC using the pre-amplified samples. Ct normalization was performed by subtracting the mean values of the housekeeping genes *Gapdh* and *Actb* from the genes of interest. A heat map and principal component analysis of the −ΔC_T_ values were plotted using the Qlucore software (*p* <0.05, q <0.2).

### Statistics

Statistical analysis was performed using SPSS version 15.0 and GraphPad Prism 5. The Mann-Whitney *U* test was used for comparisons between two groups, and the Kruskal-Wallis test was used for comparisons of more than two groups in parallel. For correlation analysis, the Spearman rank correlation coefficient was calculated for categorical variables and the Pearson correlation coefficient was calculated for continuous variables. *p* values below 0.05 (two-tailed) were considered statistically significant in all analyses.

Unpaired Student’s two-tailed *t* tests were performed using Prism 10 software (GraphPad), and heat map analysis was performed using Glucore Omics Explorer 3.5. Statistical significance was denoted as follows: ∗*p* ≤0.05; ∗∗*p* ≤0.01; ∗∗∗*p* ≤0.001; and not significant, *p* >0.05.

Additional methods are described in the supplementary materials and methods.

## Results

### HF-HCD induces a mild-to severe-MASH phenotype in wild-type C57BL/6 mice

**Fig. 1:**
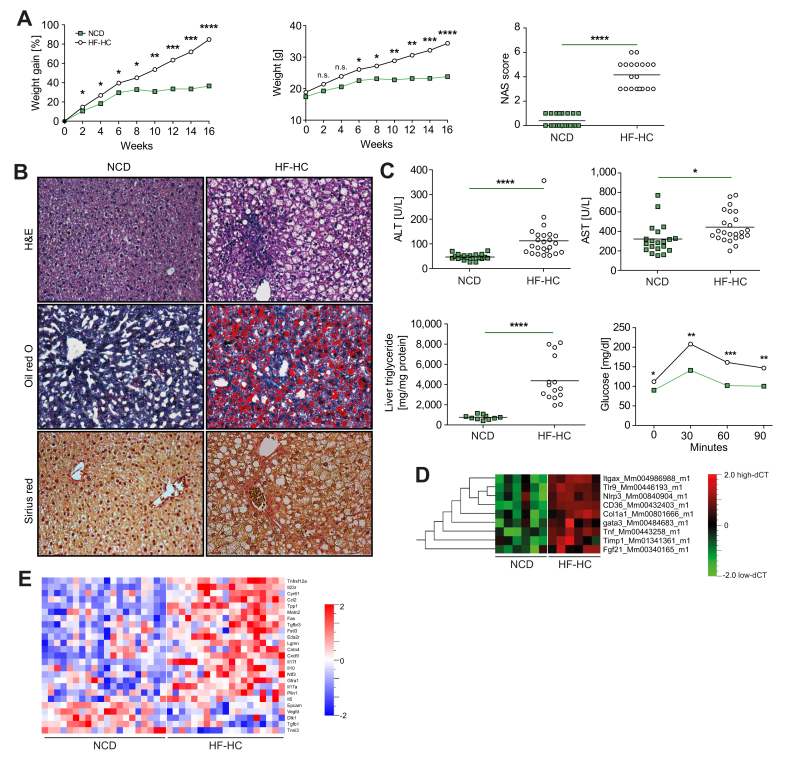
Development of MASH in C57BL/6 mice fed an HF-HCD. (A) Longitudinal weight gain in the NCD- (n = 24) and HF-HC-fed (n = 20) mice expressed as a percentage change (left panel; *p* = 0.0295, *p* = 0.0186, *p* = 0.0135, *p* = 0.0114, *p* = 0.0023, *p* = 0.0006, *p* = 0.0002, *p <*0.0001) and as absolute body weight over a 16-week period (middle panel; *p* = 0.305, *p* = 0.0953, *p* = 0.0809, *p* = 0.0413, *p* = 0.0407, *p* = 0.0066, *p* = 0.0013, *p* = 0.0006, *p <*0.0001). NAS after 16 weeks of dietary intervention (right panel; *p <*0.0001). (B) Representative H&E, ORO, and Sirius red staining of liver sections from mice fed NCD and HF-HCD showing lymphocytic infiltration, hepatocyte ballooning, and lipid and collagen accumulation in the liver of HF-HC-fed mice. (C) Quantification of serum ALT (*p <*0.0001) and AST (*p* = 0.0206) levels (upper panels), liver triglyceride levels (*p <*0.0001) and IPGTT (lower panels; *p* = 0.0227, *p* = 0.0031, *p* = 0.0002, *p* = 0.0037) in mice fed NCD and HF-HCD. (D) Differential expression of genes in the liver tissues of mice fed NCD and HF-HCD. (E) Differential expression of proteins in the sera of NCD- (n = 20) and HF-HCD-fed (n = 20) C57BL/6 mice (*p* = 0.012; q = 0.05). Unpaired two-tailed *t* tests for diet comparisons within genotype unless stated. Significance: ∗*p <*0.05, ∗∗*p* <0.01, ∗∗∗*p* <0.001, ∗∗∗∗*p* <0.0001. ALT, alanine aminotransferase; AST, aspartate aminotransferase; HE, hematoxylin and eosin; HF-HCD, high-fat/high-carbohydrate diet; IPGTT, intraperitoneal glucose tolerance test; MASH, metabolic dysfunction-associated steatohepatitis; NAS, NAFLD activity score; NCD, normal chow diet; ORO, Oil Red O.

Sixteen weeks of constant *ad libitum* feeding of HF-HCD led to significantly greater weight gain and MASH development in C57BL/6 mice, as assessed by NAS (Fig. 1A). H&E, Oil red O, and Sirius red staining of paraffin-embedded liver sections showed infiltration of inflammatory cells, increased steatosis, characteristic hepatocyte ballooning, and mild collagen deposition in HF-HCD (Fig. 1B). In line with this observation, HF-HC-fed C57BL/6 mice showed elevated aspartate and alanine aminotransferase levels, increased liver triglyceride content, and insulin resistance in the intraperitoneal glucose tolerance test (Fig. 1C).

To better characterize the pro-inflammatory response observed in the histological analysis, we performed qPCR analysis of liver tissues and evaluated the expression of genes associated with fibrosis, inflammation, T-cell homeostasis, endoplasmic reticulum stress, fatty acid metabolism, inflammasomes, and reactive oxygen species pathways (see the gene list in Table S1). Compared to NCD-fed mice, HF-HC-fed C57BL/6 mice showed an upregulation in the expression of genes belonging to T_H_1, T_H_2, and T_H_17 responses, inflammation, and inflammasome pathways (*Itgax, Gata3, Nlpr3, Tnf-α,* and *Tlr9),* fatty acid metabolism (*Fgf21* and *CD36*), and fibrosis (*Col1a1* and *Timp1)* (Fig. 1D).

To investigate the systemic levels of inflammation, we analyzed 92 serum proteins associated with responses to stress, cell motility, apoptosis, immune response, and metabolism, using Olink technology. A total of 25 proteins were differentially regulated in the serum of HF-HC-fed mice compared to NCD-fed mice (Fig. 1E). Two main clusters were identified, corresponding to proteins that were upregulated or downregulated in response to the dietary intervention. Consistent with liver inflammation, the serum levels of pro-inflammatory proteins, including CCL2, CXCL9, IL-17a, IL-17f, and TNF receptor superfamily member 12a, were increased in HF-HC-fed mice. In addition, the serum levels of the anti-inflammatory cytokine IL-10 were elevated in these mice.

### Absence of adaptive immunity in *Rag*-knockout mice barely affects the development of MASH

**Fig. 2:**
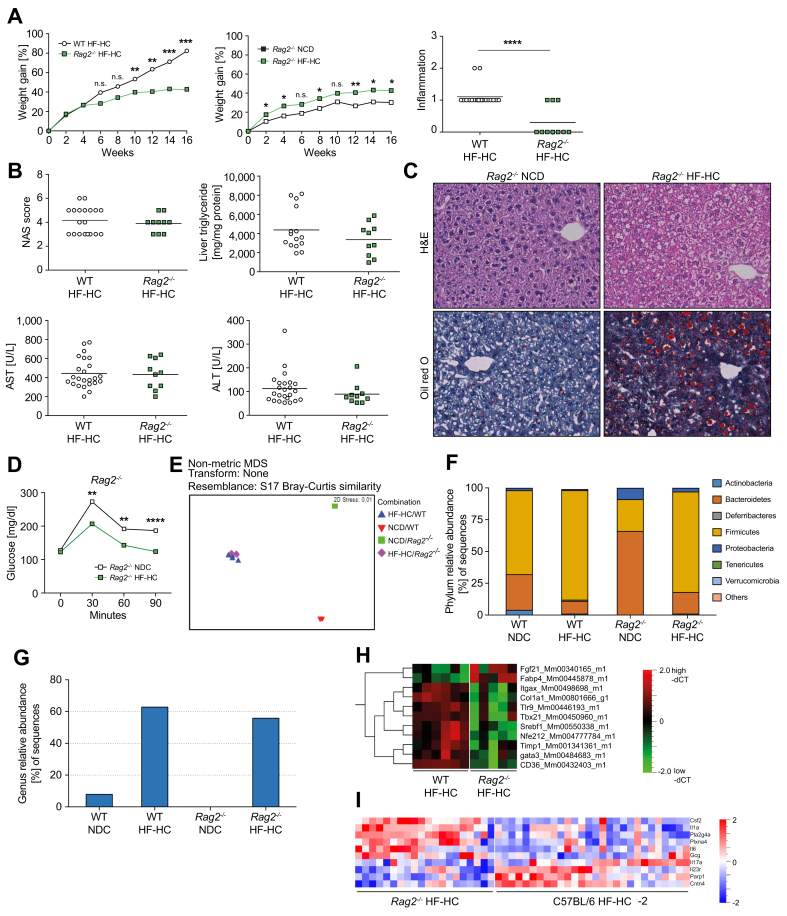
MASH development is independent of adaptive immune system. (A) Longitudinal body weight gain in the *Rag2*^−/−^ C57BL/6 (n = 10) and WT HF-HC-fed mice (as Fig. 1A, n = 20) (left panel; *p* = 0.46, *p* = 0.95, *p* = 0.073, *p* = 0.081, *p* = 0.038, *p* = 0.0034, *p* = 0.01, *p* = 0.0005) and *Rag2*^−/−^ C57BL/6 HF–HC–fed (n = 10) and NCD-fed mice (n = 10) (middle panel; *p* = 0.036, *p* = 0.015, *p* = 0.053, *p* = 0.037, *p* = 0.22, *p* = 0.0036, *p* = 0.014, *p* = 0.013) expressed as a percentage change. Liver infiltration (right panel; *p* < 0.0001) in WT (n = 20) and *Rag2*^−/−^ C57BL/6 (n = 10) mice fed an HF-HCD for 16 weeks. (B) NAS (*p* = 0.52) and liver triglyceride levels (*p* = 0.23) in HF-HC-fed WT and *Rag2*^−/−^ C57BL/6 mice (top panels). Quantification of serum ALT (*p* = 0.87) and AST levels (*p* = 0.16) (lower panel). (C) Representative H&E and ORO staining of liver sections from *Rag2*^−/−^ C57BL/6 mice fed NCD and HF-HCD showing lymphocytic infiltration, hepatocyte ballooning, and lipid accumulation in the livers of HF-HC-fed mice. (D) IPGTTs in NCD- (n = 5) and HF-HC-fed (n = 9) *Rag2*^−/−^ C57BL/6 mice (*p* = 0.65, *p* = 0.0073, *p* = 0.0071, *p* <0.0001). (E) Non-metric MDS analysis (based on the Bray-Curtis similarity index) comparing the microbial community structure in stool samples from WT and *Rag2*^−/−^ mice fed NCD and HF-HCD. (F) Relative abundance of bacterial phyla in feces in stacked bars. (G) Relative abundance of the genus *Allobaculum* in feces. (H) Differential gene expression in the liver tissue of WT (n = 6) and *Rag2*^−/−^ (n = 6) C57BL/6 mice fed HF-HCD. (I) Differential expression of proteins in the sera of WT (n = 28) and *Rag2*^−/−^ (n = 20) C57BL/6 mice fed HF-HCD (*p* = 0.005; q = 0.05). Unpaired two-tailed *t* tests for diet comparisons within genotype unless stated. Significance: ∗*p <*0.05, ∗∗*p* <0.01, ∗∗∗*p* <0.001, ∗∗∗∗*p* <0.0001. ALT, alanine aminotransferase; AST, aspartate aminotransferase; HF-HCD, high-fat/high-carbohydrate diet; IPGTT, intraperitoneal glucose tolerance test; MASH, metabolic dysfunction-associated steatohepatitis; NAS, NAFLD activity score; NCD, normal chow diet; MDS, multidimensional scaling; WT, wild-type.

To specifically analyze the role of the adaptive immune response in the induction of MASH, C57BL/6 *Rag2* knockout (*Rag2*^*-/-*^*)* mice lacking B, T, and NKT cells were fed HF-HCD. Despite the lower weight gain and inflammation grade in the liver, HF-HC-fed *Rag2*^*-/-*^ mice showed little difference in the severity of MASH compared to wild-type animals, as determined by NAS, liver triglyceride, and serum aminotransferase levels (Fig. 2A,B). Histological analysis also showed hepatocyte ballooning and lipid accumulation in the liver of HF-HC-fed *Rag2*^*-/-*^mice (Fig. 2C). In addition, HF-HC-fed wild-type and *Rag2*^*-/-*^ mice showed similar levels of glucose intolerance (Fig. 2D).

Diet reshapes the gut microbiome and influences insulin resistance and MASH susceptibility. Fecal samples from wild-type and *Rag2*^-/-^ mice fed NCD and HF-HCD revealed the predominant influence of diet on microbiome composition by deep sequencing of the 16S ribosomal DNA V1–V2 region and non-metric multidimensional scaling. As previously reported, adaptive immunity influences the microbiome composition of NCD-fed animals, as wild-type and *Rag2*^*-/-*^ mice segregated from each other in non-metric multidimensional scaling analysis. In contrast, mice fed with HF-HCD were grouped in a cluster and segregated from NCD-fed mice, regardless of their genetic background (Fig. 2E). Therefore, the influence of HF-HCD on microbiome composition is stronger than that on adaptive immunity. Changes in the microbiome of HF-HC-fed mice were marked by an expansion of Firmicutes and a reduction in Bacteroidetes (Fig. 2F). The expansion of Firmicutes can be largely attributed to the spread of the genus *Allobaculum*, which accounts for over 50% of all *Firmicutes* in mice of both genotypes fed HF-HCD (Fig. 2G). Increased numbers of *Allobaculum* are associated with higher expression of angiopoietin-like-4 (ANGPTL4) and hepatocyte-specific depletion of *Angptl4* protects mice from diet-induced obesity, glucose intolerance, and hepatic steatosis. Therefore, changes in the microbiome caused by HF-HCD consumption appear to be sufficient to induce MASH in mice lacking adaptive immune responses, even though the inflammatory status of the liver differs between the wild-type and *Rag2*^−/−^ mice.

Molecular analysis of liver tissue reflected differences in inflammation due to the absence of an adaptive immune system. While HF-HC-fed wild-type mice showed increased expression of genes related to T-cell responses (*Itgax, Gata3, Tbx21,* and *Tlr9)* and fibrosis (*Col1a1* and *Timp1)*, genes related to the regulation of glucose and lipid metabolism (*Fgf21* and *Fabp4*) were upregulated in *Rag2*^*-/-*^ mice (Fig. 2H). Serum protein levels reflected these differences, with wild-type mice showing increased T-cell response markers (IL-17a, sIL-23R, PARP1, and CNTN4) and *Rag2*^−/−^ mice exhibiting elevated levels of the pro-inflammatory cytokines IL-1a and IL-6 (Fig. 2I).

Taken together, our data suggest that adaptive immunity influences the severity of liver inflammation but not the development of MASH.

### Innate immune responses in wild-type and *Rag*-knockout mice fed HF-HCD

In our model, *Rag2*^−/*−*^ mice fed HF-HCD developed MASH despite the absence of adaptive immune responses. Innate immune cells are implicated in MASH progression. Therefore, we quantified Kupffer cells (KCs), monocyte-derived macrophages (MoMFs) and neutrophils.

**Fig. 3:**
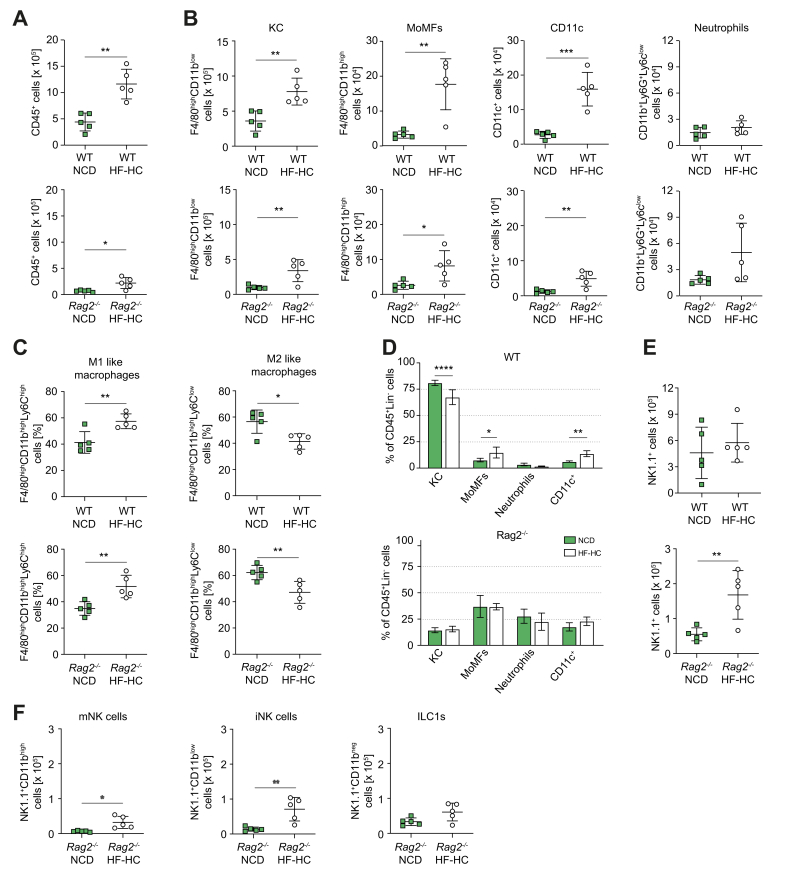
The role of innate immunity in MASH development: Quantitative analysis of flow cytometry data of innate immune cells in the livers of wild-type and *Rag2*^-/-^ C57BL/6 mice fed NCD and HF-HCD. (A) The total number of Lin-CD45^+^ cells (WT *p* = 0.0012; *Rag2*^−/−^*p* = 0.014). (B) Total number of resident Kupffer cells (KC; WT *p* = 0.0045, *Rag2*^−/−^*p* = 0.0099), monocyte-derived macrophages (MoMFs; WT *p* = 0.0024, *Rag2*^−/−^*p* = 0.024), CD11c^+^ cells (WT *p* = 0.0003, *Rag2*^−/−^*p* = 0.0058), and neutrophils (WT *p* = 0.22, *Rag2*^−/−^*p* = 0.072). (C) Frequency of pro-inflammatory M1-like (WT *p* = 0.0068, *Rag2*^−/−^*p* = 0.0056) and anti-inflammatory M2-like macrophages (WT *p* = 0.013, *Rag2*^−/−^*p* = 0.0096). (D) Frequency distribution of KC, MoMFs, CD11c^+^ cells, and neutrophils in the livers of wild-type and *Rag2*^−/−^ mice (one-way ANOVA *p* <0.0001; Tukey *post hoc*). (E) Total number of intrahepatic NK1.1^+^ cells in WT and *Rag2*^−/−^ mice (WT *p* = 0.50; *Rag2*^−/−^*p* = 0.008). (F) Subpopulations of NK1.1^+^ cells in the livers of *Rag2*^−/−^ mice (mNK *p* = 0.0112; iNK *p* = 0.0057, ILC1s *p* = 0.056). Unpaired two-tailed *t* tests for diet comparisons within genotype unless stated; bars show mean ± SD; n = 5 mice per group. Significance: ∗*p <*0.05, ∗∗*p* <0.01, ∗∗∗*p* <0.001, ∗∗∗∗*p* <0.0001. HF-HCD, high-fat/high-carbohydrate diet; iNK, immature natural killer cell; ILC1, innate lymphoid cell type 1; KC, Kupffer cell; Lin, lineage; MoMF, monocyte-derived macrophage; mNK, mature natural killer cell; NCD, normal chow diet; NK, natural killer cell; WT, wild-type.

Although more cells were found in wild-type livers, HF-HC-fed mice of both genotypes showed increased total CD45^+^ cells (Fig. 3A), mainly because of elevated levels of KCs, MoMFs, and CD11c+ cells (Fig. 3B).

MoMFs are further subdivided into pro-inflammatory M1-like and anti-inflammatory M2-like macrophages. The number of M1-like macrophages increased, while that of M2-like macrophages decreased in both genotypes when fed a HF-HCD (Fig. 3C). Plasmacytoid dendritic cells (DCs), conventional type 1 DCs, conventional type 2 DCs, and monocytes increased in HF-HC-fed mice, regardless of the genotype (Fig. S1A). Infiltrating MoMFs and CD11c^+^ cells increased significantly in wild-type livers, but not in *Rag2*^-/-^ livers, correlating with decreased KCs (Fig. 3D). Notably, the percentage of liver infiltrating cells (neutrophils, MoMFs, and monocytes) in *Rag2*^-/-^ mice fed NCD was already higher than that in wild-type mice and was comparable to or higher than that in wild-type mice fed a HF-HCD (Fig. S1B).

The hepatic NK1.1^+^ population includes conventional immature NK cells, mature NK cells, and innate lymphoid cells 1. In our model, the total number of NK1.1^+^ cells was significantly increased in the livers of *Rag2*^-/-^ but not wild-type mice (Fig. 3E). This increase was due to an increase in the number of immature and mature NKs, but not innate lymphoid cells 1 (Fig. 3F).

Overall, the innate immune response to HF-HCD was similar to BALB/c, suggesting that T cells play a pivotal role in disease progression and severity.

### Strong activation and clonal expansion of intrahepatic T cells in mice fed HF-HCD

To investigate the involvement of T cells in inducing and exacerbating MASH, we analyzed the T-cell compartments of wild-type mice fed NCD or HF-HCD.

**Fig. 4:**
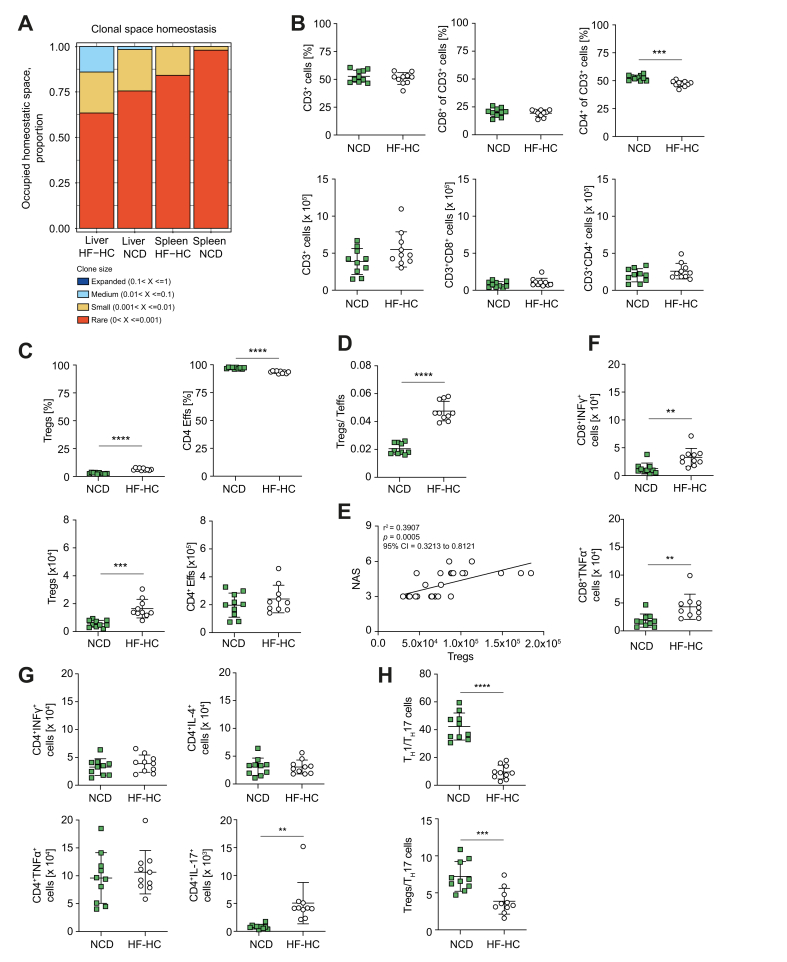
T-cell effector and regulatory responses in C57BL/6 mice fed an HF-HCD. (A) Expansion of T-cell clones in the livers of HF-HC-fed mice. (B) Frequency and total number of intrahepatic CD3+ (frequency *p* = 0.41, abs. *p* = 0.099), CD8+ (frequency *p* = 0.60, abs. *p* = 0.18), and CD4+ T cells (frequency *p* = 0.0001, abs. *p* = 0.22), and (C) Tregs (frequency *p* <0.0001, abs. *p* = 0.0001) and CD4+ Teffs (frequency *p* <0.0001, abs. *p* = 0.30) in NCD- and HF-HC-fed mice. (D) Increased Treg/T effector ratio in the liver of HF-HC-fed mice (*p* <0.0001). (E) Correlation between Treg number and MASH severity. (F, G) Expression of pro-inflammatory cytokines by intrahepatic CD8+ (IFN-γ *p* = 0.0035; TNF-α *p* = 0.0066) and CD4+ T cells (IFN-γ *p* = 0.38; TNF-α *p* = 0.59; IL-4 *p* = 0.94; IL-17 *p* = 0.002) in NCD- and HF-HC-fed mice. (H) Predominant T_H_17 response in mice fed the HF-HCD (T_H_1/T_H_17 *p* <0.0001; Tregs/T_H_17 *p* = 0.0008). Unpaired two-tailed *t* tests for diet comparisons within genotype unless stated. Data from one representative experiment are presented (n = 10 per group). Significance: ∗*p <*0.05, ∗∗*p* <0.01, ∗∗∗*p* <0.001, ∗∗∗∗*p* <0.0001. HF-HCD, high-fat/high-carbohydrate diet; HF-HC, high-fat/high-carbohydrate; MASH, metabolic dysfunction-associated steatohepatitis; Teff, T effector cell; T_H_, T helper; Treg, regulatory T cell.

Our analysis of the ß-TCR repertoire revealed a notable expansion of T-cell clones in the livers of mice fed with HF-HCD, indicating localized antigen-driven activation. Although these clones represented up to 20% of all intrahepatic T cells in the HF-HCD group, their presence was minimal in mice on the NCD, accounting for less than 5% of T cells (Fig. 4A). Nonetheless, no differences were observed in the proportions and absolute numbers of CD3^+^ and CD8^+^ T cells. However, there was a distinct decrease in the proportion of CD4^+^ T cells in mice fed a HF-HCD, accompanied by a significant increase in the relative and absolute numbers of intrahepatic Tregs (Fig. 4B,C). This increase was accompanied by a significant decrease in the proportion of CD4^+^ effector T cells, whereas the total number of these cells remained unchanged (Fig. 4C). The increase in Tregs resulted in a significant shift in the Treg/Teff ratio towards Tregs (Figs 4D and S2A). However, Tregs appeared to have a deleterious effect on MASH because the number of intrahepatic Tregs was positively correlated with NAS (Fig. 4E).

To test this possibility, we treated HF-HCD-fed mice with low doses of anti-CD3 F(ab)2' fragments to expand Tregs. Although this treatment significantly increased the proportion of Tregs and intrahepatic Treg/Teff ratio (Fig. S2B), it failed to affect body weight, glucose metabolism, or NAS (Fig. S2C). Moreover, intrahepatic triglyceride levels were notably elevated posttreatment (Fig. 2D).

Despite the higher Treg/Teff ratio, the proportion and number of activated CD8^+^ T cells producing inflammatory cytokines were significantly increased in the livers of wild-type mice fed a HF-HCD (Figs 4F and S3A,B). This discrepancy suggests compromised regulation of CD8^+^ effector T cells by Tregs in metabolically inflamed livers. Unexpectedly, transferring CD8^+^ T cells into *Rag2*^-/-^ mice lacking Tregs and feeding them a HF-HCD resulted in milder disease outcomes than in wild-type mice, despite increased intrahepatic CD8^+^ effector T cells expressing pro-inflammatory cytokines (Fig. S3C,D). This implies that the deleterious effects of Tregs are unlikely to be mediated by CD8^+^ T-cell dysregulation.

Intrahepatic accumulation of T_H_17 cells has been identified as a contributing factor in the development and progression of MASH. Moreover, HF-HCD feeding significantly increased the number of intrahepatic CD4^+^ T cells expressing IL-17, but not IFN-γ, TNF-α, or IL-4, indicating a pro-inflammatory T_H_17 response in MASH (Fig. 4G,H). This shift towards a T_H_17-dominated environment in the liver of mice with MASH was further supported by the transfer of CD4^+^ T cells into *Rag2*^-/-^ mice on a HF-HCD, which resulted in a marked increase in intrahepatic T_H_17 cells accompanied by a decrease in the number of Tregs (Fig. S4). This suggests that T_H_17 cells may serve as key mediators of liver inflammation in HF-HC-fed mice.

### Lobular accumulation of Tregs increases with MASLD severity in patients

In mice fed a HF-HCD, we observed a positive correlation between the number of intrahepatic Tregs and NAS. To test whether this is also the case in humans, we analyzed the infiltration of CD4^+^ and CD8^+^ effector T cells and Tregs in liver biopsies from 32 patients diagnosed with MASLD. Patients were divided into two groups according to the AASLD guidelines: MASLD without MASH and MASLD with MASH. Twenty-three of these patients met the criteria for MASH, while nine did not (Table S2). Most patients were pre-obese, had additional hyperlipoproteinemia (MASLD, 4/9; MASH, 13/23), and no or mild liver fibrosis.

**Fig. 5:**
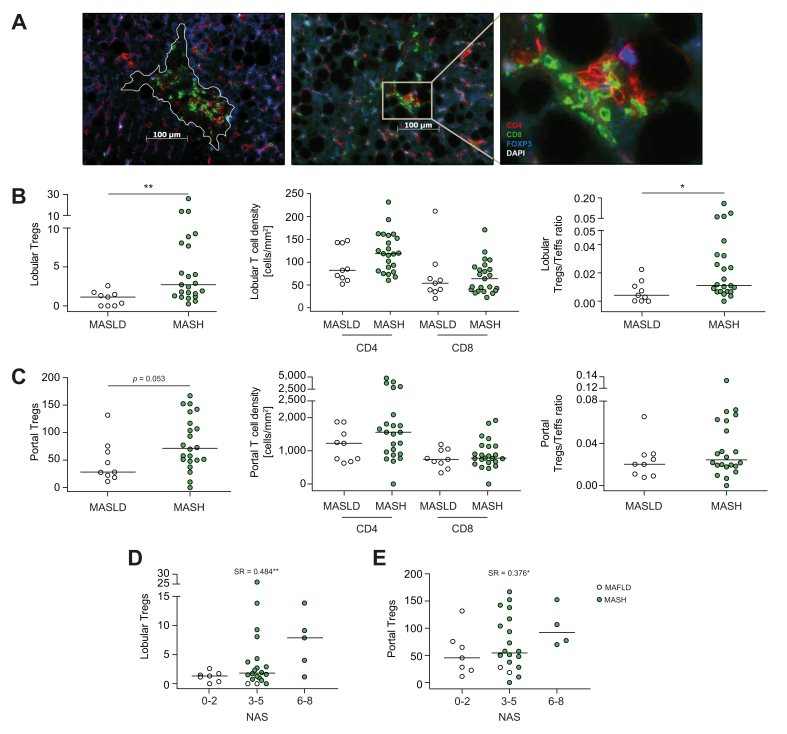
The accumulation of Tregs is correlated with disease severity in patients with MASH. (A) Immunofluorescence staining for CD8 (green), CD4 (red), and FOXP3 (blue) in paraffin sections from patients with MASH. (B, C) Quantitative analysis of stained T-cell populations in patients with MASLD and MASH. (B) Lobular areas (Tregs *p* = 0.0042*;* CD4 *p* = 0.094; *CD8 p* = 0.65; ratio *p* = 0.015); (C) Portal areas (Tregs *p* = 0.053; CD4 *p* = 0.198; *CD8 p* = 0.386; ratio *p* = 0.379). (D, E) Correlation analysis between the Treg numbers and NAS. (D) Lobular areas; (E) Portal areas (MASLD, n = 9; MASH, n = 23). Mann-Whitney *U* test unless stated; Significance: ∗*p <*0.05, ∗∗*p* <0.01, ∗∗∗*p* <0.001, ∗∗∗∗*p* <0.0001. MASH, metabolic dysfunction-associated steatohepatitis; MASLD, metabolic dysfunction-associated steatotic liver disease; NAS, NAFLD activity score; Treg, regulatory T cell.

To investigate the correlation between Treg levels and disease severity, biopsy sections were stained for CD4, CD8, and FOXP3 (Fig. 5A). Portal and lobular T-cell infiltrates were also evaluated. Consistent with murine data, lobular infiltration of Tregs was denser in biopsy sections from patients with MASH. In contrast, the densities of CD4^+^ and CD8^+^ effector T cells were similar in both groups, resulting in a significant increase in Treg/Teff ratio in patients with MASH (Fig. 5B). In the portal tracts, the number of Tregs also increased in patients with MASH but did not reach significance (Fig. 5C). Notably, the accumulation of Tregs, but not CD4^+^ or CD8^+^ T cells, increased with the histological severity of MASLD in both portal tracts and liver lobules and correlated with NAS (Figs 5D,E and S5A,B). However, the Treg/Teff ratio correlated with NAS only in lobular areas (Fig. 5E). This correlation was significant for steatosis and hepatocellular ballooning but not for lobular inflammation (Fig. S5C). Therefore, our results demonstrated that intrahepatic Tregs also accumulate in patients with MASH. Importantly, the density of Treg infiltration correlated with the severity of the MASH scores. Taken together, these data indicate that Tregs play a crucial role in MASH pathology.

## Discussion

In this study, 16 weeks of HF-HC feeding resulted in marked steatohepatitis, fibrosis and metabolic dysfunction in C57BL/6 mice. In contrast, *Rag2*^-/-^ animals lacking adaptive lymphocytes exhibited a comparable steatotic load but a significantly milder inflammatory score. These findings suggest that adaptive immunity acts to amplify, rather than initiate, disease severity. The HF-HCD induced widespread innate activation and clonal T-cell expansion; although low-dose anti-CD3 F(ab')_2_ successfully increased the intrahepatic Treg/Teff ratio, it failed to improve NAS and instead increased hepatic triglycerides, highlighting a paradoxical, disease-aggravating effect of Treg expansion in this context. Corroborating the murine observations, liver biopsies from patients with MASH displayed a higher lobular Treg density that positively correlated with NAS, thereby underscoring the translational relevance of Treg accumulation in progressive steatohepatitis.

The development of MASH is closely associated with obesity, metabolic syndrome, and insulin resistance.^2^ Animal models should, therefore, mimic these disease conditions to advance our understanding of pathophysiology and help develop new therapeutic interventions. The steatosis and inflammation observed with methionine- or choline-deficient diets lack key disease features, and even combinations of toxic and high-fat diets may promote inflammation but do not replicate the MASH phenotype seen in patients.^22^ Therefore, a HF-HCD, including fructose, is currently one of the best ways to induce the MASH phenotype in otherwise healthy inbred mouse strains.^22^^,^^23^

Both innate and adaptive immunity are involved in the pathology of MASH.^8^ In this study, we showed that 16 weeks of HF-HCD feeding was sufficient to induce MASH in both C57BL/6 wild-type and *Rag2*^*-/-*^ mice, with knockout animals exhibiting a slightly milder grade of liver inflammation. Therefore, we did not observe a lack of steatosis, intrahepatic inflammation, or MASH as reported by Wolf *et al.* in C57BL/6 *Rag1*^*-/-*^ mice.^12^ This discrepancy is unlikely to be due to genotype differences between *Rag1* and *Rag2* knockout mice, as HF-HC-fed BALB/c *Rag1*^*-/-*^ mice developed more severe disease^13^ than our C57BL/6 *Rag2*^*-/-*^ mice. As Wolf *et al.* used an MCD-HFD, this discrepancy is most likely due to differences in the diets used in the studies. In fact, HF-HCD induced a much more severe metabolic syndrome in a shorter period, indicating a stronger effect of HF-HCD on lipid metabolism. In addition, our data showed that the induction of glucose intolerance was not dependent on adaptive immune cells. Consistent with the data described for BALB/c *Rag1*^*-/-*^ mice,^13^ we also observed a significant increase in intrahepatic NK cells and M1 macrophages in C57BL/6 *Rag2*^*-/-*^ mice fed an HF-HCD.

In addition to macrophages, NK1.1^+^ cells are also involved in the induction of MASH, although their role in the development and progression of the disease is not fully understood.^4^ Mature NKs produce high levels of IFN-γ,^21^ which enhances M1 macrophage differentiation, exacerbates inflammation, and attenuates fibrosis.^24^ This may explain the lack of collagen deposition in the livers of *Rag2*^−/−^ mice fed a HF-HCD. Other pro-inflammatory cytokines secreted by these cells, such as TNF-α and IL-1, can inhibit insulin signaling,^25^ leading to the severe phenotype observed in knockout animals. Our results support the idea that the innate immune response may be more important than the adaptive immune response in the development of insulin resistance.^13^

Only 10-20% of MASLD cases progress to MASH, which suggests that genetic factors, in addition to environmental factors, influence disease predisposition and progression. Dietary composition can alter the gut microbiome, leading to changes in insulin resistance and MASH development in mice^26^ and humans.[27], [28], [29] Under chow conditions, Rag-deficiency alters microbial communities;^30^ our data confirm this point. In the present study, HF-HCD led to expansion of the phylum Firmicutes. Previous studies demonstrated an association between an increased Firmicutes/Bacteroidetes ratio and obesity.^27^ In addition, the strong expansion of the genus *Allobaculum*, which is known to induce the upregulation of ANGPTL4, a protein associated with diet-induced obesity, glucose intolerance, and hepatic steatosis,^31^^,^^32^ may support the development of MASH independent of innate immunity. Therefore, our data suggest that a lack of adaptive immunity has less influence on the induction of MASH than the dietary regimen, indicating that genetic factors other than adaptive immunity are involved in MASH predisposition. However, even if the adaptive immune response is not an essential component of MASH development, the milder degree of inflammation observed in *Rag2*^*-/-*^ mice suggests that the adaptive immune response increases disease severity.

CD8^+^ T cells play a pivotal role in driving MASH progression by releasing pro-inflammatory molecules and contributing to hepatocyte damage.^6^^,^^8^ Consistent with this observation, an accumulation of intrahepatic CD8^+^ T cells correlating with disease progression has been observed in several mouse models and in patients.^12^^,^^33^^,^^34^ In contrast, Ma *et al.* observed no changes in the number of intrahepatic CD8^+^ T cells but a decrease in the number of CD4^+^ T cells.^11^ In all of these studies, changes in the T-cell compartment resulted in a decreased intrahepatic CD4^+^/CD8^+^ T-cell ratio in the inflamed liver. In our study, we did not observe any significant changes in the absolute numbers of CD8^+^ and CD4^+^ T cells, either in the experimental model or in patients with MASH. Thus, we could not confirm the reduced CD4^+^/CD8^+^ T-cell ratio observed in previous studies.^11^^,^^12^^,^^33^ This discrepancy may be because our study included patients with less fibrosis than other studies. However, it is noteworthy that in a study by Ma *et al.*, the CD4^+^/CD8^+^ T-cell ratio was only reduced compared to hepatitis B and C controls but not compared to normal controls and patients with alcohol-related steatohepatitis.^11^ The discrepancy observed in mouse data is most likely due to differences in diet and study design. A recent meta-analysis categorized over 3,900 unique rodent models of MASH, highlighting the high heterogeneity of study designs and the need for standardization and replication. Even studies that appeared to use the same model, that is, the same diet and genetic background, were so different that they were considered unique in the meta-analysis.^23^ This heterogeneity complicates the validation and comparison of preclinical data.

The role of CD4^+^ T cells in MASH pathology is largely dependent on their phenotype and function, and many aspects of the CD4^+^ T-cell response in MASH remain to be elucidated.^6^^,^^8^ Wolf *et al.* reported an increase in CD4^+^ T-cell numbers in mice fed an MCD-HFD.^12^ In contrast, Ma *et al.* observed a relative and absolute decrease in CD4^+^ T-cell counts in MASLD and HCC models associated with feeding MCD-HFD or choline-deficient and amino acid-deficient diets. This decrease was due to a reduction in T_H_1 effector cells and Tregs, while the absolute number of T_H_17 cells increased.^11^ In our study, we also observed a decrease in the proportion of intrahepatic CD4^+^ T cells in mice fed a HF-HCD, but the absolute numbers of these cells remained unchanged. In agreement with the data published by Wang *et al.*,^35^ we found a strong increase in the relative and absolute numbers of intrahepatic Tregs both in the mouse model and in patients with MASH. Additionally, elevated serum levels of IL-10 and soluble IL-23R suggest modulation of systemic inflammation, as IL-10 and sIL-23R regulate T_H_1 and T_H_17 responses.^36^

Despite the increased Treg/Teff ratio, a higher number of CD8^+^ T cells expressing pro-inflammatory cytokines was observed in the livers of HF-HC-fed mice, indicating impaired regulation of CD8^+^ T cells by Tregs. However, the transfer of CD8^+^ T cells into *Rag2*^-/-^ mice fed HF-HC resulted in a further increase in the number of intrahepatic pro-inflammatory CD8^+^ T cells, suggesting that Tregs partially counterbalanced T-cell activation in wild-type mice. Preferential or supra-proportionate intrahepatic accumulation of Tregs, correlating with disease severity, has also been observed in patients with other types of hepatitis such as acute and subclinical liver allograft rejection and autoimmune hepatitis.^15^^,^^37^ One potential explanation for the persistent inflammation under investigation is the ability of neutrophil extracellular traps (NETs) to activate CD4+ T cells.^35^ Despite the absence of an assessment of NET formation in the present study, this pathway warrants investigation in future experiments.

However, what is the significance of the increased Treg/Teff ratio in MASH? In liver transplantation, an increased FOXP3^+^/CD8^+^ ratio in the liver is associated with poorer graft survival,^38^ whereas in kidney transplantation, higher Treg/CD8^+^ ratios in the grafts are associated with fewer subsequent rejections and better 5-10 year survival.^39^ In this context, it may seem paradoxical that patients with MASH have higher Treg levels than those without. However, the pattern of immune regulation observed in our cohort of patients with MASLD and early-stage MASH (Table S2) could explain why only a minority of the patients progressed to advanced cirrhosis and HCC. In contrast, increased Treg/Teff ratios could contribute to the development of liver cancer in patients in the premalignant stages of MASH.^35^

Although the therapeutic potential of Tregs in several diseases is undoubted,^40^ the accumulation of Tregs in the liver does not resolve inflammation in mice or patients with MASH. Others have described the control of adipose tissue inflammation and improvement of insulin sensitivity in mice fed a HFD.^41^ In contrast, we found that the number of intrahepatic Tregs positively correlated with MASH severity in both mice and humans. Furthermore, increasing intrahepatic Treg numbers by anti-CD3 F(ab)2' fragment therapy did not improve disease in C57BL/6 mice but worsened disease in BALB/c mice.^13^ Consequently, elevated levels of aminotransferases and Tregs imply a probable disease-promoting effect of Tregs, which is in accordance with earlier findings in alternative mouse models.^13^^,^^35^ Contrary to our expectations, CD8^+^ T cell transfer in HF-HC-fed *Rag2*^-/-^ mice resulted in amelioration, but not exacerbation, of disease, suggesting that these cells are not the major effector cell population involved in MASH pathology. This may explain why Tregs failed to control liver inflammation. Intriguingly, the transfer of Tregs in BALB/c *Rag1* ^*−/−*^ mice exacerbates MASH.^13^ As these mice lack T and B cells, either the interaction between Tregs and cells of the adaptive immune system promotes inflammation or Tregs differentiate into CD4^+^ effector T cells in the inflamed liver.

Intrahepatic accumulation of T_H_17 cells has also been implicated in the development and progression of MASH in mice and humans.^9^^,^^10^ Tregs and T_H_17 cells share TGF-β as a developmental factor, and under pro-inflammatory conditions, Tregs can differentiate into T_H_17-like cells, altering the T_H_17/Treg balance.^42^ In addition, metabolic factors, such as fatty acid metabolism and glycolysis, as well as gut microbiota, can influence the T_H_17/Treg balance and favor T_H_17 differentiation.^43^ In MASH, accumulation of T_H_17 cells in the liver is associated with disease exacerbation, most likely due to increased production of IL-17A.^9^^,^^10^^,^^35^ Notably, intrahepatic accumulation of T_H_17 cells was observed even when the total number of CD4^+^ T cells decreased.^11^ In our experiments, HF-HC feeding increased the number of intrahepatic T_H_17 cells in wild-type mice and resulted in preferential development of T_H_17 cells in the livers of *Rag2*^-/-^ recipients after CD4^+^ T-cell transfer. Interestingly, the increase in the number of IL-17-expressing cells was accompanied by a decrease in the number of Tregs. Therefore, we propose a model in which CD4^+^ T cells infiltrating the liver differentiate into Tregs because of their interactions with NETs. With increasing metabolic inflammation, a subset of these Foxp3^+^ cells gain IL-17A expression, contributing to disease exacerbation. Lineage-tracing studies will be required to confirm any true fate conversion of Tregs into T_H_17 cells.

The present investigation is subject to several limitations. Firstly, CD8-cell depletion was not performed; while informative, such an intervention would require additional large cohorts and currently lacks a therapeutically applicable counterpart in patients. Secondly, lineage-tracing experiments were not conducted to ascertain whether Foxp3+ IL-17A+ cells derive from stable Tregs or former effector cells. Thirdly, NET formation and function were not analyzed, which means that the contribution of extracellular traps is only speculative. In conclusion, the HF-HCD mouse model has been demonstrated to be a valuable tool in the study of human MASH, capturing numerous aspects of the condition. However, it is imperative to exercise caution when translating these findings to the clinical setting.

Effective therapeutic interventions for MASLD other than lifestyle changes are just beginning to enter clinical practice. The translation of immunological findings into therapeutic development is undoubtedly hampered by the lack of reproducibility of MASH mouse models and different cohorts of patients analyzed in different studies. However, the data indicate that the T-cell subsets that infiltrate the metabolically inflamed liver are highly plastic and may act in different ways than expected depending on the degree and type of inflammation. Therefore, the effects of immunomodulatory therapies may vary depending on disease stage. This information is important for designing future therapeutic trials for treating patients with MASH.

## Abbreviations

ANGPTL4, angiopoietin-like 4; DC, dendritic cell; HCC, hepatocellular carcinoma; HF-HC, high-fat/high-carbohydrate; MASH, metabolic dysfunction-associated steatohepatitis; MASLD, metabolic dysfunction-associated steatotic liver disease; NAS, NAFLD activity score; NCD, normal chow diet; SBP, spontaneous bacterial peritonitis; Teff, effector T cell; T_H_, T helper cell; Tregs, regulator T cells.

## Financial support

This work was supported by grants from the German Research Foundation (KFO250 project 7; SFB TR 127 project A4; BU 2722/2-3, and HA 6880/2-3), the Integrated Research Center Transplantation (IFB-Tx projects CBT3, ISI5, and ISI6) funded by the German Federal Ministry of Education and Research (reference number: 01EO1302 and 01EO0802), the Government of Canada's New Frontiers in Research Fund (NFRF), NFRFT-2020-00787, and the European Union’s Horizon 2020 Research and Innovation Program (825392; RESHAPE consortium). A.K.B. was supported by the M.D. dissertation program (StrucMed).

## Authors’ contributions

Study concept and design: RT, EJ, and MHW; acquisition of data: JD, LEBM, AKB, CMH, FN, ACDM, KLHB, MS, JS, CSF, MB, HB, CK, FW, BH, and MB; analysis and interpretation of data: JD, LEBM, AKB, ACDM, RT, MHW; drafting and writing of the manuscript: LEBM, ACDM, RT, MHW; critical revision of the manuscript for important intellectual content: all; statistical analysis: JD, LEBM, ACDM, SR, BH, RT; obtained funding: LEBM, AKB, FN, RT, EJ, MHW; administrative, technical, or material support: MS, JS, CK, HB, MB, MPM, and HW; and study supervision: RT, EJ, MHW.

## Data availability

The datasets generated and/or analyzed during the present study are available from the corresponding authors upon reasonable, scientifically justified request.

## Conflict of interest

The authors declare no conflicts of interest.

Please refer to the accompanying ICMJE disclosure forms for further details.
