# Supplementary material for: From model to man: Understanding Tregs' dual role in MASLD
Source: JHEP Rep. 2025 Oct 9;7(12):101619. doi: 10.1016/j.jhepr.2025.101619 (PMC12682130; doi:10.1016/j.jhepr.2025.101619)
Supplement: Multimedia component 1 [file mmc1.pdf]

From model to man: Understanding Tregs' dual role in MASLD

Janine Dywicki, Laura Elisa Buitrago-Molina, Anna K. Baumann, Ana C. Davalos-Misslitz, Celina M. Hendriks, Katharina L. Hupa-Breier, Maren Lieber, Jerome Schlue, Matthias Blüher, Heike Bantel, Christine S. Falk, Christian Koenecke, Freya Wellhöner, Benjamin Heidrich, Michael P. Manns, Fatih Noyan, Heiner Wedemeyer, Richard Taubert, Elmar Jaeckel, Matthias Hardtke-Wolenski

Table of contents

Supplementary materials and methods…………………………………………………….. 2

Table S1…………………………………………………………………………………...4

Fig. S1……………………………………………………………………………………..6

Fig. S2……………………………………………………………………………………..7

Fig. S3……………………………………………………………………………………..8

Fig. S4……………………………………………………………………………………..9

Fig. S5……………………………………………………………………………………10

Fig. S6……………………………………………………………………………………11

Table S1…………………………………………………………………………………12

Supplementary references……………………………………………………………..13

Supplementary materials and methods

Liver Triglyceride Measurements

Frozen murine liver tissue was homogenized in a standard diluent (Cayman) with 20 µg/ml leupeptin (Serva) via a rod homogenizer for 10-15 s and centrifuged. Colorimetric detection and quantitation of total protein was performed using the Pierce™ BCA Protein Assay Kit (Thermo Fisher Scientific). Triglycerides in the supernatant were detected via a Cobas® 8000 modular analyzer (HITACHI/Roche).

Flow Cytometry

Mice organs were minced, and intrahepatic lymphocytes (IHLs) were separated using a 40%/70% Percoll (GE Healthcare) gradient. Mice splenic red blood cells were lysed, and lymphocytes were subsequently stained with appropriate combinations of anti-CD3, anti-CD4, anti-CD8, anti-CD25, anti-Ki-67, anti-B220, anti-Foxp3, anti-CD62L, anti-CD49b, anti-NK1.1, anti-Ly6G, anti-Ly6C, anti-CD45, anti-CD11b, anti-F4/80, anti-CD11c, anti-TNF-α and anti-IFN-γ antibodies. All acquisitions were performed with an LSRII SORP interfaced with DIVA software (BD Biosciences).

T cell repertoire

Cell isolation and cell sorting

Cell isolation from spleen and liver from mice were redundant to flow cytometry preparations. CD4 and CD8 positive cells were isolated like in cell separation and adoptive transfer and pooled afterwards.

Nucleic acid isolation and RACE

Total RNA was isolated from MACSed cell populations using the RNeasy Mini Kit (Qiagen). To generate libraries of murine CDR3 *beta* regions, anchor sequence-containing cDNA template was synthesized by using the SMARTer RACE cDNA Amplification Kit (Clontech) according to the manufacturer guidelines. Subsequent PCR was performed with a TCR *beta* chain gene-specific 3' primer located at a conserved gene-site of the constant region. According to manufacturer's protocol, we used an anchor sequence specific 5' universal primer mix (UPM) to amplify all full-length cDNA products. The UPM included a custom modified primer with Illumina adapter sequences added to it. PCR products were purified using the gel extraction kit QIAquick Gel Extraction (QIAGEN) according to manufacturer guidelines and were further quantified via the NanoDrop 1000 Spectrophotometer (Thermo Fisher Scientific).

High-throughput sequencing and Sequence analysis

High-throughput sequencing was done on an Illumina MiSeq system using the MiSeq Reagent Kit v3 600-cycle). Sequence analysis of murine CDR3 *beta* regions was done using a bioinformatics pipeline established by the Institute of Immunology at Hannover Medical School. Raw sequence data is first run through a quality filter (http://www.bioinformatics.babraham.ac.uk/projects/fastqc/) that removes low-quality reads. Subsequently, files were aligned to a reference genome using the web tool IMGT/HighV-QUEST[1, 2]. After alignment, in-house written program scripts that pipeline the analysis of sequence data were used; these include the programs VDJtools[3], tcR R-package [4] and currently in-house program scripts for statistical analysis.

Microbiome analysis

Sample acquiring

Feces from mice were collected separately and immediately frozen on dry ice and stored at -80 °C until samples are validated.

Further sample handling and analysis (DNA extraction, sequencing, bioinformatical and statistical analysis) was done as described in [5] by Heidrich *et al.*.

Protein Detection in the Serum by Olink

In brief, pairs of oligonucleotide-labeled antibody probes bind to their targeted protein, and if the two probes are brought in close proximity the oligonucleotides will hybridize in a pair-wise manner. The addition of a DNA polymerase leads to a proximity-dependent DNA polymerization event, generating a unique PCR target sequence. The resulting DNA sequence is subsequently detected and quantified using a microfluidic real-time PCR instrument (Biomark HD, Fluidigm). Data is then quality controlled and normalized using an internal extension control and an inter-plate control, to adjust for intra- and inter-run variation. The final assay read-out is presented in Normalized Protein eXpression (NPX) values, which is an arbitrary unit on a log2-scale where a high value corresponds to a higher protein expression. All assay validation data (detection limits, intra- and inter-assay precision data, etc.) are available on manufacturer's website (www.olink.com).

Immunofluorescence

Immunofluorescence microscopy was performed as described previously[6]. In brief, cryo sections of murine tissue were rehydrated; blocked; stained with anti-murine-CD68, anti-murine-F4/80, and anti-murine-CD11b antibodies and DAPI; and analyzed with AxioImagerM1 using AxioVision 4.8 software (Zeiss).

TaqMan® Assays

All TaqMan® Assays for murine transcripts were purchased from Thermo Fisher Scientific. Assays are tagged with FAM and MGB and have murine specificity.

Table S1: (murine mRNAs)

gene symbol	Assay ID	Gene name
Nfe2l2	Mm00477784_m1	Nuclear factor (erythroid-derived 2)-like 2
CD36	Mm00445273_m1	CD36
Tlr4	Mm00445273_m1	Toll-like receptor 4
Tnf	Mm00443258_m1	Tumor necrosis factor alpha
Srebf1	Mm00550338_m1	Sterol regulatory element binding transcription factor 1
Tlr9	Mm00446193_m1	Toll-like receptor 9
Il1b	Mm00434228_m1	Interleukin 1 beta
Nlrp3	Mm00840904_m1	NLR family, pyrin domain containing 3
Fabp4	Mm00445878_m1	Fatty acid binding protein 4, adipocyte
Fgf21	Mm00340165_g1	Fibroblast growth factor 21
Timp1	Mm01341361_m1	Tissue inhibitor of metalloproteinase 1
Ccl2	Mm00441242_m1	Chemokine (C-C motif) ligand 2
TbX21	Mm00450960_m1	T-box 21
Foxp3	Mm00475162_m1	Fork head box P3
Il17a	Mm00439618_m1	Interleukin 17A
Col1a1	Mm00801666_g1	Collagen, type I, alpha 1
Itgax	Mm00498698_m1	Integrin alpha X
Mmp2	Mm00439498_m1	Matrix metallopeptidase 2
Tgfb2	Mm00436955_m1	Transforming growth factor, beta 2
Acta2	Mm00725412_s1	Actin, alpha 2, smooth muscle, aorta
Il23	Mm01160011_g1	Interleukin 23
Gapdh	Mm99999915_g1	Glyceraldehyde-3-phosphate dehydrogenase
Actb	Mm00607939_s1	Actin, beta
Gata3	Mm00484683_m1	GATA binding protein 3

Cell Separation and Adoptive Transfer

Biotin-conjugated monoclonal antibodies against murine $CD4^+$ or $CD8^+$ (both homemade) were used for magnetic cell sorting of mice splenocytes. Streptavidin Microbeads (Miltenyi) were used as described in the manufacturer's guidelines. A total of $5*10^6$ $CD4^+$ or $CD8^+$ cells/animal was injected intravenously.

Anti-CD3 F(ab)2' fragments therapy

Mice fed the NCD and HF-HC diets were injected intraperitoneally with fifty micrograms of anti-CD3 F(ab)2' (145-2C11, Bio-X-Cell) or isotype control (Bio-X-Cell) for five consecutive days

during week 17 of feeding. Mice were maintained on the diet until the end of the experiment and analyzed at week 22.

Fig. S1

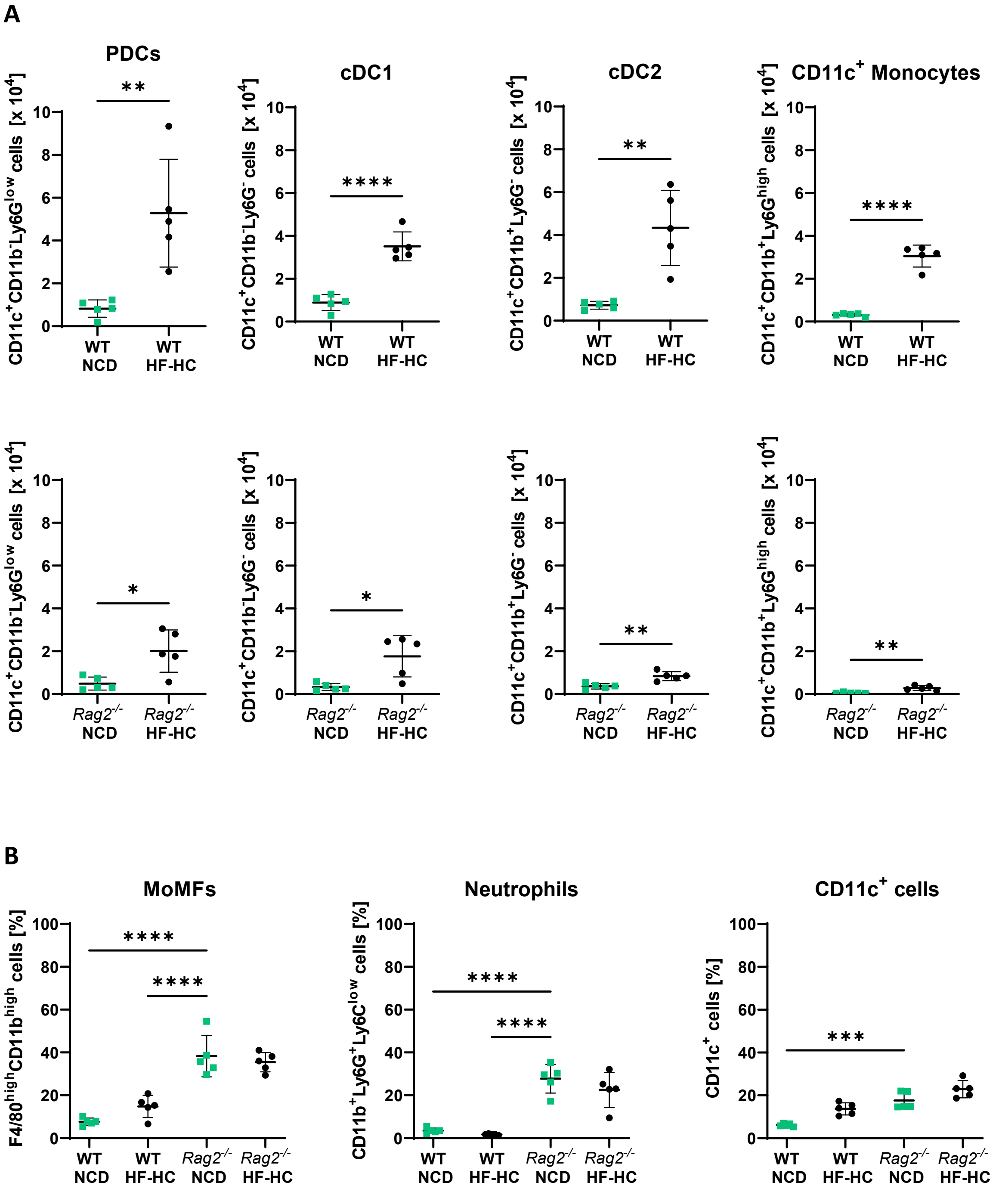

Fig. S1. Innate immune cell populations in the liver of WT and Rag2$^{-/-}$ C57BL/6 mice fed NCD and HF-HC diet. (A) CD11c^{+} cell subpopulations. **(B)** Proportions of MoMFs, Neutrophils and CD11c^{+} cells in the liver of WT and Rag2$^{-/-}$ mice under the same dietary conditions. Shown are data from one experiment, n=5 per group. Means ±SD; unpaired t-test **(A)**; one-way ANOVA followed by Šídák's multiple comparisons test **(B)**; *P< 0.05, **P< 0.01, ***P< 0.001.

Fig. S2

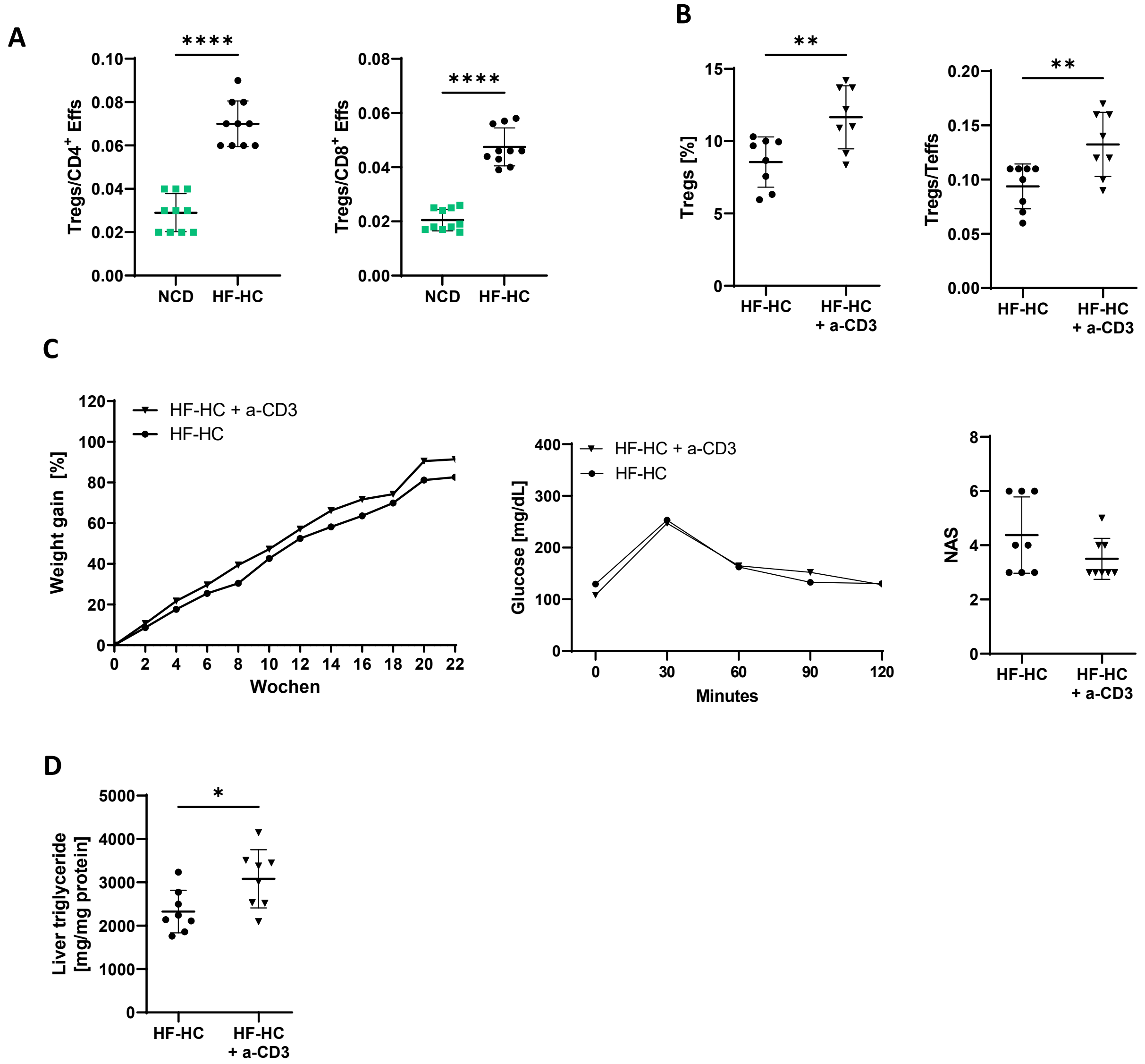

Fig. S2. Tregs are unable to control HF-HC diet induced metabolic inflammation. (A) Increased Treg/Teff ratio for CD4⁺ and CD8⁺ effector T cells in the liver of wild-type C57Bl/6 mice fed the HF-HC diet. **(B)** Expansion of Tregs after therapy with anti-CD3 F(ab)2' fragments. **(C, D)** Expansion of Tregs does not lead to disease improvement. Shown are data from one experiment, n=5 per group. Means ±SD; unpaired t-test; *P< 0.05, **P< 0.01, ****P< 0.0001.

Fig. S3

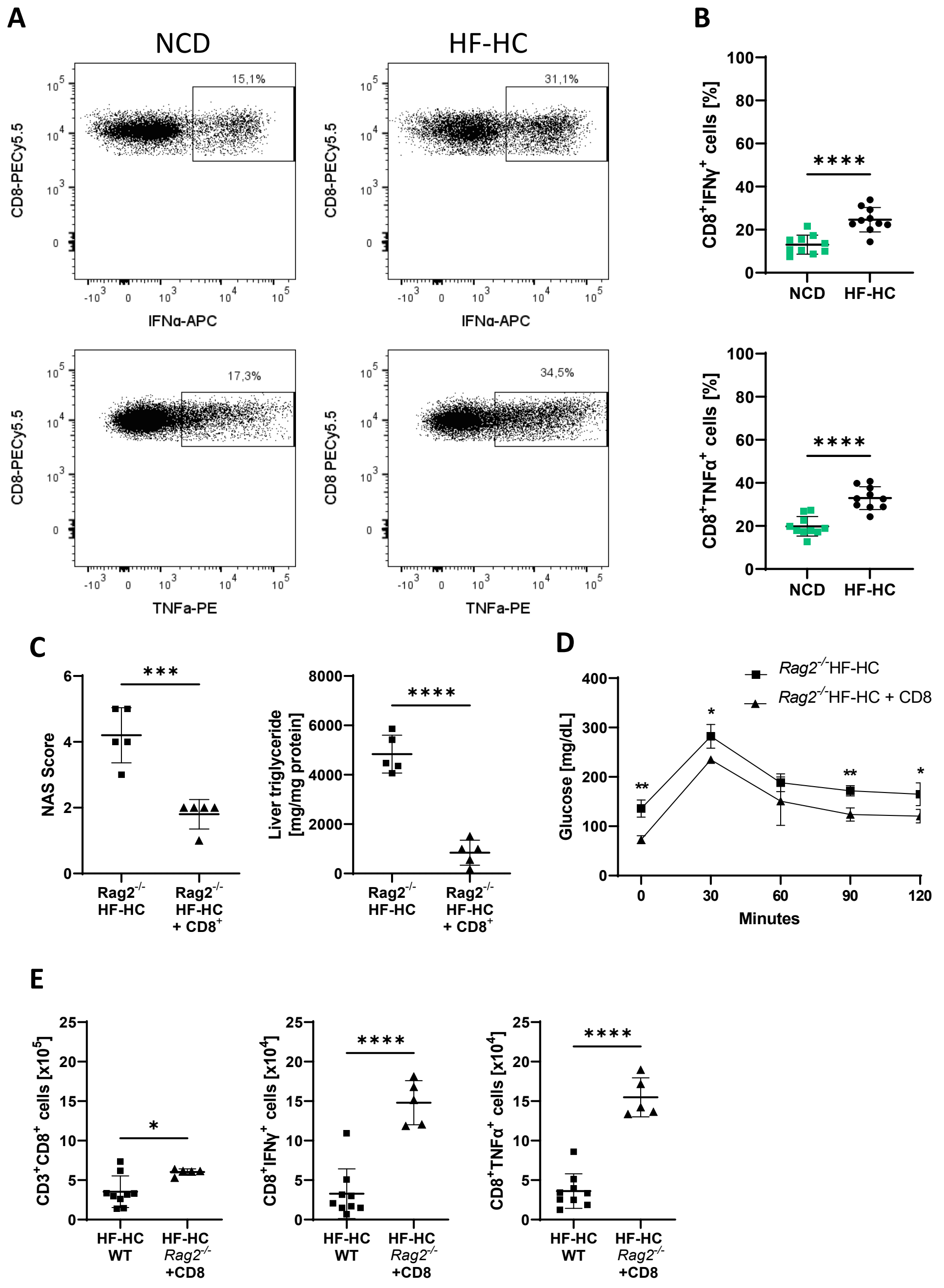

Fig. S3. T cell responses induced by HF-HC diet. (A, B) Expression of pro-inflammatory cytokines by CD8$^+$ T cells in the liver of NCD and HF-HC fed wild-type C57BL/6 mice. Representative data from one of 2 experiments, n=10 per experiment. **(C, D)** NAS score, hepatic triglyceride levels and glucose intolerance test of Rag2$^{-/-}$ mice after CD8$^+$ T cell transfer compared to wild-type mice. **(E)** Total number of intrahepatic CD8$^+$ T cells expressing inflammatory cytokines in the same animals. Shown are data from a transfer experiment in Rag2$^{-/-}$ mice, n=5, compared to a representative experiment in wild-type mice, n=10. Means ±SD; unpaired t-test.

Fig. S4

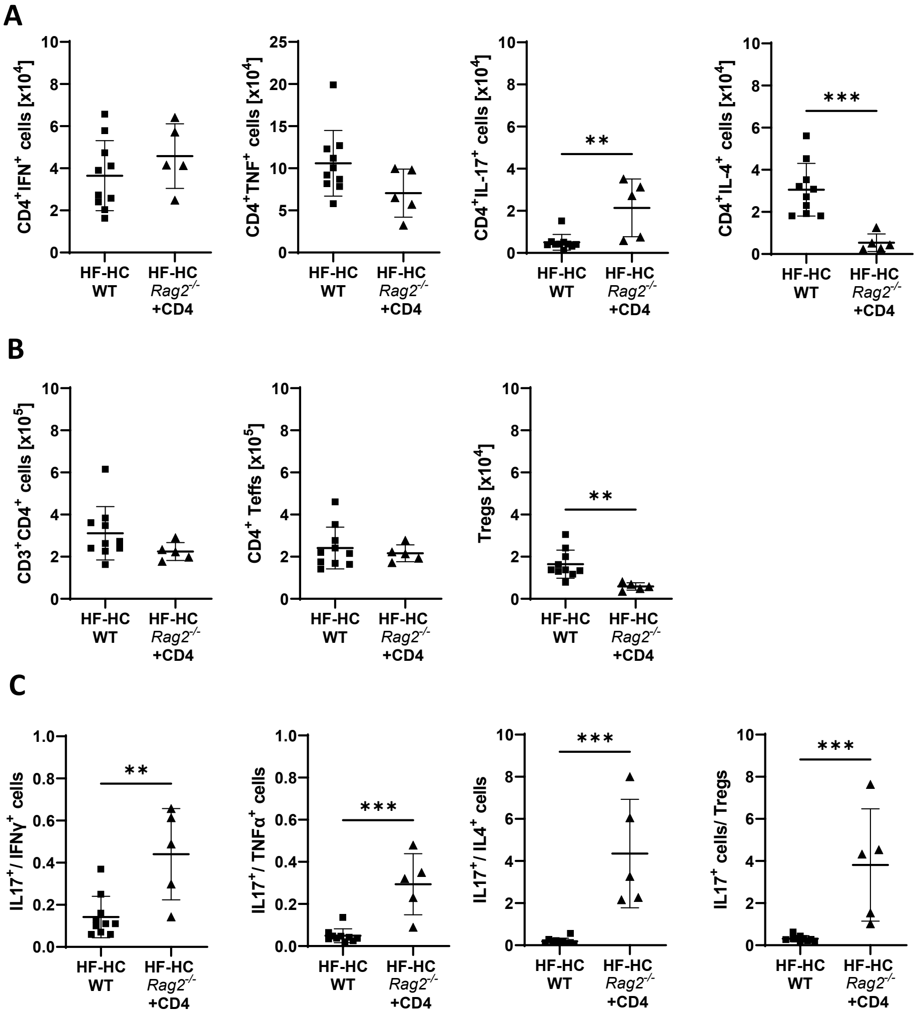

Fig. S4. Preferential differentiation of transferred CD4+ T cells into T_H17-like cells in the liver of $Rag2^{-/-}$ mice fed HF-HC diet. CD4+ T cells were transferred into $Rag2^{-/-}$ mice 6 weeks after starting the HF-HC diet and mice were sacrificed 10 weeks later. **(A)** Cytokine expression of intrahepatic CD4+ T cells showing an increase in the number of cells expressing IL-17 and a decrease in the number of cells expressing IL-4 in $Rag2^{-/-}$ recipient mice. **(B)** Decreased number of intrahepatic Tregs **(C)** Predominance of the T_H17 response in $Rag2^{-/-}$ recipient mice. Shown are data from a transfer experiment in $Rag2^{-/-}$ mice, n=5, compared to a representative experiment in wild-type mice, n=10. Means ±SD; unpaired t-test.

Fig. S5.

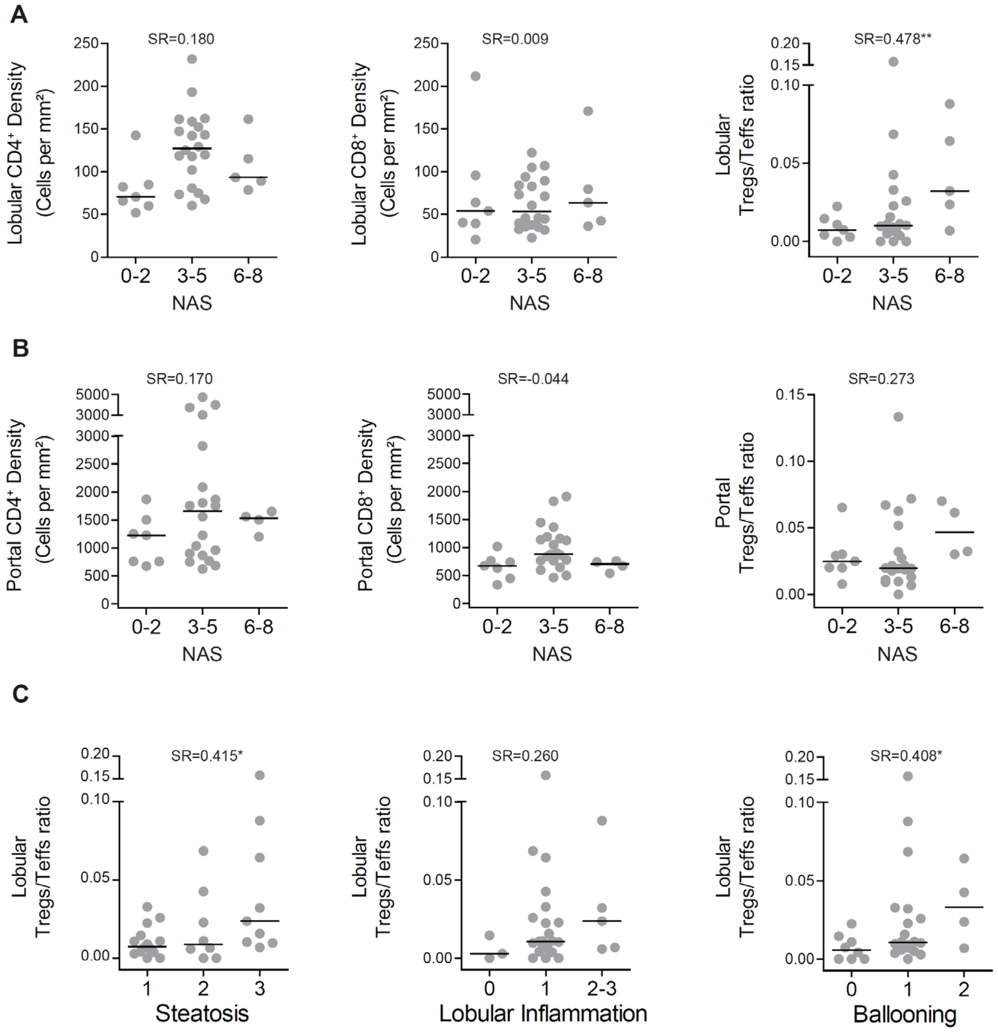

Fig. S5. Correlation between regulatory T cell numbers and disease severity in NASH patients. (A, B) Correlation between Tregs/Teffs ratio and NAS score in lobular but not in portal areas of the liver. **(C)** Correlation between lobular Tregs/Teffs ratio and steatosis and ballooning. n=32. Spearman rank correlation coefficient (SR). *P< 0.05, **P< 0.01, ***P< 0.001.

Fig. S6

Fig. S6. Adaptive immune cell populations in the liver of WT C57BL/6 mice fed NCD and HF-HC diet. Flow cytometry data of HF-HC (n=20) or NCD (N=20) fed mice showing **(A)** B220$^+$, **(B)** CD3$^+$, and **(C)** CD3$^+$CD8$^+$ cell subpopulations. All were not significantly changed. **(D)** Hepatic cryosection of HF-HC fed mouse stained for CD4 (green), CD8 (blue), and Foxp3 (red). **(E)** Representative flow cytometric plot of HF-HC fed murine liver, gated on CD3$^+$CD4$^+$ shows CD4 vs IL-17A.

Table S2

Patient data

	**MASLD without MASH** median (IQR)	N	**MASH** median (IQR)	N
Age at biopsy (years)	35.0 (27.0)	9	42.0 (20.0)	23
Gender (male/female)	7/2		16/7	
BMI (kg/m²)	26.0 (4.7)	9	26.5 (4.6)	23
**Laboratory Tests**				
Fasting Glucose (mM)	4.7 (0.9)	6	4.4 (0.6)	15
Insulin (µU/ml)	17.4 (16.5)	6	20.5 (21.9)	15
HOMA	3.4 (4.2)	6	3.9 (3.5)	15
M30 (U/l)	170.2 (-)	3	476.4 (547.9)	13
Leptin (ng/ml)	6.1 (-)	3	6.0 (7.9)	13
Adiponectin (µg/ml)	9.7 ()	3	8.3 (2.7)	13
Cholesterol (mg/dl)	236.0 (47.0)	9	213.1 (66.0)	23
Triglyceride (mg/dl)	137.0 (114.0)	9	171.0 (128.0)	23
VLDL C. (mg/dl)	23.0 (16.0)	9	25.0 (25.0)	23
LDL C. (mg/dl)	149.0 (47.0)	9	143.0 (57.0)	23
HDL C. (mg/dl)	55.0 (13.0)	9	40.0 (16.0)	23
Alanine aminotransferase (U/l)	58.0 (39.0)	8	120.0 (74.0)	23
Aspartate aminotransferase (U/l)	34.0 (8.0)	7	63.0 (43.0)	22
Glutamate dehydrogenase (U/l)	10.0 (2.3)	4	12.0 (10.3)	10
Gamma-glutamyl transferase (U/l)	144.0 (254.5)	9	112.0 (146.0)	23
Alkaline phosphatase (U/l)	104.5 (69.3)	8	78.0 (41.0)	17
Bilirubin (µM)	11.0 (14.0)	9	11.5 (8.8)	22
**Histology**				
NAS-Score	2.0 (1.0)	9	4.0 (2.0)	23
Steatosis	1.0 (0.5)	9	2.0 (2.0)	23
Lobular Inflammation	1.0 (1.0)	9	1.0 (0.0)	23
Ballooning	0.0 (0.0)	9	1.0 (0.0)	23
Fibrosis (Ishak)	0 (0.0)	9	0 (1.0)	22
**Follow up**				
Duration of follow up	59.6 (36.4)	8	57.8 (35.4)	22
Liver enzyme elevation (normalization/improved/stable)	1/0/7	8	5/4/13	22
Last non-invasive fibrosis measurement (Metavir)	<F2 (0.0)	8	<F2 (1.0)	14

Supplementary references

1. Alamyar, E., P. Duroux, M.P. Lefranc, et al., *IMGT((R)) tools for the nucleotide analysis of immunoglobulin (IG) and T cell receptor (TR) V-(D)-J repertoires, polymorphisms, and IG mutations: IMGT/V-QUEST and IMGT/HighV-QUEST for NGS.* Methods Mol Biol, 2012. **882**: p. 569-604.
2. Li, S., M.P. Lefranc, J.J. Miles, et al., *IMGT/HighV QUEST paradigm for T cell receptor IMGT clonotype diversity and next generation repertoire immunoprofiling.* Nat Commun, 2013. **4**: p. 2333.
3. Shugay, M., D.V. Bagaev, M.A. Turchaninova, et al., *VDJtools: Unifying Post-analysis of T Cell Receptor Repertoires.* PLoS Comput Biol, 2015. **11**(11): p. e1004503.
4. Nazarov, V.I., M.V. Pogorelyy, E.A. Komech, et al., *tcR: an R package for T cell receptor repertoire advanced data analysis.* BMC Bioinformatics, 2015. **16**: p. 175.
5. Heidrich, B., M. Vital, I. Plumeier, et al., *Intestinal microbiota in patients with chronic hepatitis C with and without cirrhosis compared with healthy controls.* Liver Int, 2017.
6. **Hardtke-Wolenski, M.**, **K. Fischer, F**. Noyan, et al., *Genetic predisposition and environmental danger signals initiate chronic autoimmune hepatitis driven by CD4+ T cells.* Hepatology, 2013. **58**(2): p. 718-28.
