# Supplementary material for: From model to man: Understanding Tregs' dual role in MASLD
Source: JHEP Rep. 2025 Oct 9;7(12):101619. doi: 10.1016/j.jhepr.2025.101619 (PMC12682130; doi:10.1016/j.jhepr.2025.101619)
Supplement: Multimedia component 2 [file mmc2.docx]

**JHEP Reports**

**CTAT methods**

Tables for a “Complete, Transparent, Accurate and Timely account” (CTAT) are now mandatory for all revised submissions. The aim is to enhance the reproducibility of methods.

- Only include the parts relevant to your study
- Refer to the CTAT in the main text as ‘Supplementary CTAT Table’
- Do not add subheadings
- Add as many rows as needed to include all information
- Only include one item per row

**If the CTAT form is not relevant to your study, please outline the reasons why:**

|  |
| --- |

- 1. **Antibodies**

| **Name** | **Citation** | **Supplier** | **Cat no.** | **Clone no.** |
| --- | --- | --- | --- | --- |
| anti-CD3 |  | Biolegend |  | 17A2 |
| anti-CD4 |  | Biolegend |  | GK1.5 |
| Anti-CD8 |  | Biolegend |  | 53-5.8 |
| Anti-CD25 |  | Biolegend |  | PC61 |
| Anti-Ki-67 |  | Biolegend |  | 11F6 |
| Anti-B220 |  | Biolegend |  | RA3-6B2 |
| Anti-Foxp3 |  | Biolegend |  | MF-14 |
| Anti-CD62L |  | Biolegend |  | MEL-14 |
| Anti-CD49b |  | Biolegend |  | HMa2 |
| Anti-NK1.1 |  | Biolegend |  | S17016D |
| Anti-Ly6G |  | Biolegend |  | 1A8 |
| Anti-Ly6C |  | Biolegend |  | HK1.4 |
| Anti-CD45 |  | Biolegend |  | 30-F11 |
| Anti-CD11b |  | Biolegend |  | M1/70 |
| Anti-F4/80 |  | Biolegend |  | QA17A29 |
| Anti-TNFa |  | Biolegend |  | MP6-XT22 |
| Anti-IFNg |  | Biolegend |  | XMG1.2 |
| Anti-IL4 |  | Biolegend |  | 11B11 |
| Anti-IL17A |  | Biolegend |  | TC11-18H10.1 |
| Anti- CD3 F(ab)2' |  | Bio-X-Cell |  | 145-2C11 |
| anti-CD68 |  | Biolegend |  | FA-11 |

- 1. **Cell lines**

| **Name** | **Citation** | **Supplier** | **Cat no.** | **Passage no.** | **Authentication test method** |
| --- | --- | --- | --- | --- | --- |
| **na** |  |  |  |  |  |

- 1. **Organisms**

| **Name** | **Citation** | **Supplier** | **Strain** | **Sex** | **Age** | **Overall n number** |
| --- | --- | --- | --- | --- | --- | --- |
| C57Bl/6J |  | Jackson | C57Bl/6J | Male and female | 6-8 weeks |  |
| C57Bl/6 Rag2^-/-^ |  | Jackson | C57BL/6-*Rag2^tm1Cgn^*/J | Male and female | 6-8 weeks |  |

- 1. **Sequence based reagents**

| **Name** | **Sequence** | **Supplier** |
| --- | --- | --- |
| **na** |  |  |

- 1. **Biological samples**

| **Description** | **Source** | **Identifier** |
| --- | --- | --- |
| **na** |  |  |

- 1. **Deposited data**

| **Name of repository** | **Identifier** | **Link** |
| --- | --- | --- |
| **na** |  |  |

- 1. **Software**

| **Software name** | **Manufacturer** | **Version** |
| --- | --- | --- |
| Prism 10 for macOS | GraphPad | 10.4.2 |
| FlowJo | Becton Dickinson & Company | 10.10.0 |
| AxioVision | Zeiss | 4.8 |
|  |  |  |

- 1. **Other (*e.g*. drugs, proteins, vectors etc.)**

| **na** |  |  |
| --- | --- | --- |
|  |  |  |

- 1. **Please provide the details of the corresponding methods author for the manuscript:**

| Prof. Dr. Matthias Hardtke-Wolenski  Hannover Medical School  Dept. of Gastroenterology, Hepatology, Infectious Diseases and Endocrinology  OE6815, Carl-Neuberg-Str. 1, D-30625 Hannover  Tel.: +49 511 532 9513  Email: [Wolenski.Matthias@mh-hannover.de](mailto:Wolenski.Matthias@mh-hannover.de) |
| --- |

**2.0 Please confirm for randomised controlled trials all versions of the clinical protocol are included in the submission. These will be published online as supplementary information.**

| **na** |
| --- |
